# Identification of hub genes and establishment of a diagnostic model in tuberculosis infection

**DOI:** 10.1186/s13568-024-01691-7

**Published:** 2024-04-13

**Authors:** Chunli Liu, Xing Li

**Affiliations:** Department of Respiratory, Chongqing Dazu Traditional Chinese Medicine Hospital, No.218, 1st Ring North Road, Dazu District, Chongqing, 402360 China

**Keywords:** Tuberculosis, PPI, Diagnostic model, Immune Infiltration, Biomarkers

## Abstract

Tuberculosis (TB) poses significant challenges due to its high transmissibility within populations and intrinsic resistance to treatment, rendering it a formidable respiratory disease with a substantial susceptibility burden. This study was designed to identify new potential therapeutic targets for TB and establish a diagnostic model. mRNA expression data for TB were from GEO database, followed by conducting differential expression analysis. The top 50 genes with differential expression were subjected to GO and KEGG enrichment analyses. To establish a PPI network, the STRING database was utilized, and hub genes were identified utilizing five algorithms (EPC, MCC, MNC, Radiality, and Stress) within the cytoHubba plugin of Cytoscape software. Furthermore, a hub gene co-expression network was constructed using the GeneMANIA database. Consistency clustering was performed on hub genes, and ssGSEA was utilized to analyze the extent of immune infiltration in different subgroups. LASSO analysis was employed to construct a diagnostic model, and ROC curves were used for validation. Through the analysis of GEO data, a total of 159 genes were identified as differentially expressed. Further, GO and KEGG enrichment analyses revealed that these genes were mainly enriched in viral defense, symbiotic defense, and innate immune response-related pathways. Hub genes, including DDX58, IFIT2, IFIH1, RSAD2, IFI44L, OAS2, OAS1, OASL, IFIT1, IFIT3, MX1, STAT1, and ISG15, were identified using cytoHubba analysis of the PPI network. The GeneMANIA analysis unmasked that the co-expression rate of hub genes was 81.55%, and the physical interaction rate was 12.27%. Consistency clustering divided TB patients into two subgroups, and ssGSEA revealed different degrees of immune infiltration in different subgroups. LASSO analysis identified IFIT1, IFIT2, IFIT3, IFIH1, RSAD2, OAS1, OAS2, and STAT1 as eight immune-related key genes, and a diagnostic model was constructed. The ROC curve demonstrated that the model exhibited excellent diagnostic performance. DDX58, IFIT2, IFIH1, RSAD2, IFI44L, OAS2, OAS1, OASL, IFIT1, IFIT3, MX1, STAT1, and ISG15 were hub genes in TB, and the diagnostic model based on eight immune-related key genes exhibited good diagnostic performance.

## Introduction

Tuberculosis (TB) is a highly prevalent infectious disease caused by Mycobacterium tuberculosis (Mtb), leading to substantial morbidity and mortality worldwide. Recent data indicates that in 2020 alone, there were an estimated 9.87 million new cases of TB globally, resulting in an incidence rate of 127 per 100,000 individuals Global tuberculosis report (2021), (2021). These figures highlight the significant burden imposed on the economic and health status of affected individuals 2021 (Bagcchi 2021). The main cause of TB is the inhalation and ingestion of Mtb by alveolar macrophages, which leads to the formation of granulomas due to the interaction between Mtb and inflammatory cells (Moule and Cirillo 2020). The main clinical features of TB are long-term low fever, cough, and hemoptysis. Mtb has the capability to infect various tissues and organs across the body, encompassing the lungs, intestines, lymph nodes, joints, spine, and genitourinary system (Gopalaswamy et al. 2020). TB primarily spreads through the respiratory tract, making early and effective diagnosis and treatment pivotal in greatly reducing TB-related fatalities (Bouton and Jacobson 2021). The standard treatment for TB includes a four-drug regimen of isoniazid (INH), rifampicin (RMP), ethambutol (EMB), and pyrazinamide (PZA) for two months, followed by a two-drug regimen of RMP and INH for another four months. However, some patients develop drug resistance to at least one of the drugs, leading to adverse reactions and hepatotoxicity (Suarez et al. 2019). The identification of drug resistance and cure during treatment mainly relies on sputum culture and smear results. However, in the later stages of treatment, the reduced production of sputum in patients limits the applicability of this method (Gunther et al. 2021). Hence, the identification and development of novel biomarkers that encompass persistent pathological alterations and individual variabilities in patients hold immense significance in facilitating early diagnosis of TB and devising personalized treatment strategies.

The occurrence, development, and outcome of TB are intricately determined by the immunological recognition, response, and regulation of Mtb. When the invasiveness of Mtb is weaker than that of the host immune system, alveolar macrophages can directly kill and eliminate Mtb (Walters et al. 2021). Afterward, macrophages, NK cells, and other innate immune cell groups can generate immune memory, and the immune system can produce faster and more effective protective immune responses upon the second invasion of Mtb (Divangahi et al. 2021). When the invasiveness of Mtb reaches a balance with the host immune system, Mtb may enter a dormant state, presenting an immune escape and symbiotic state with the host (Gong and Wu 2021). When the invasiveness of Mtb is stronger than that of the host immune system, Mtb can replicate in granulomas, and granulomas may undergo caseous necrosis, liquefaction, and cavitation, leading to the spread of Mtb and the occurrence of active TB (de Martino et al. 2019). The integral role of immune responses in the immune defense against TB necessitated the exploration of immune response-derived biomarkers during Mtb infection. By establishing a diagnostic model incorporating immune-related genes, the diagnostic efficacy of TB can be enhanced, enabling the timely assessment of patients’ immune status, infection progression, and prognostic risks, thereby facilitating prompt patient triage.

In this study, we performed bioinformatics analysis on the TB gene expression profile from GEO database and identified TB hub genes through PPI network and Cytoscape analysis of these hub genes’ functions in the onset and progression of TB. Then, we screened hub genes using LASSO analysis and constructed a diagnostic model for TB, offering new ideas for TB diagnosis.

## Materials and methods

### Data collection

TB microarray datasets GSE83456 (platform: GPL10558; control: 61; TB: 92) and GSE19444 (platform: GPL6947; control: 12; TB: 21) were from GEO database (https://www.ncbi.nlm.nih.gov/) and used as the training and validation sets, respectively.

### Identification of differentially expressed genes (DEGs)

To identify DEGs between healthy individuals and TB patients, *limma* package (Ritchie, et al. 2015) was utilized to do differential expression analysis on GSE83456 dataset. DEGs were selected with a threshold of |logFC|> 1 and adjust_p < 0.05.

### Gene Ontology (GO) and Kyoto Encyclopedia of Genes and Genomes (KEGG) enrichment analyses

The top 50 genes with the highest affinity scores were chosen, and GO functional analysis and KEGG pathway enrichment analysis (*P* < 0.05) were performed using *clusterProfiler* package (Yu, et al. 2012) in R software. The results were presented with corresponding bubble charts.

### Construction of a protein–protein interaction (PPI) network

PPI network was constructed utilizing STRING database (https://string-db.org/) to analyze interactions among DEGs. Interaction relationships with confidence scores greater than or equal to 0.7 were selected.

### Screening of hub genes

The top 20 hub genes were calculated utilizing five algorithms (EPC, MCC, MNC, Radiality, and Stress) in the cytoHubba plugin of Cytoscape software. Through the utilization of Venn diagram analysis, we successfully identified a set exhibit shared presence across five algorithms.

### GeneMANIA database

The GeneMANIA (http://www.genemania.org) database was used to construct a co-expression network, provide gene function predictions, and identify genes sharing similar functions. In-depth analysis was performed on the co-expression network and the associated functions of these hub genes.

### Consensus clustering and immune infiltration analysis

The *ConsensusClusterPlus* package (Wilkerson and Hayes 2010) was employed to conduct clustering analysis on all TB patient samples, utilizing the expression levels of hub genes as the basis for clustering. Single-sample GSEA (ssGSEA) method was utilized to evaluate the level of immune infiltration in each TB patient sample, and the ssGSEA package (Barbie, et al. 2009) was used to analyze the variations in immune infiltration levels among patients in different subgroups.

### Construction and validation of the diagnostic model

The *glmnet* package (Friedman et al. 2010) was utilized to screen for key genes that affect TB and construct a LASSO analysis diagnostic model ground on the hub genes. Subsequently, *pROC* package (Robin et al. 2011) was used to plot receiver operating characteristic (ROC) curve and calculate the area under the curve (AUC) value to validate the diagnostic model. The diagnostic accuracy of the model was subsequently validated using an independent validation set.

## Results

### Differential expression analysis of TB

**Fig. 1:**
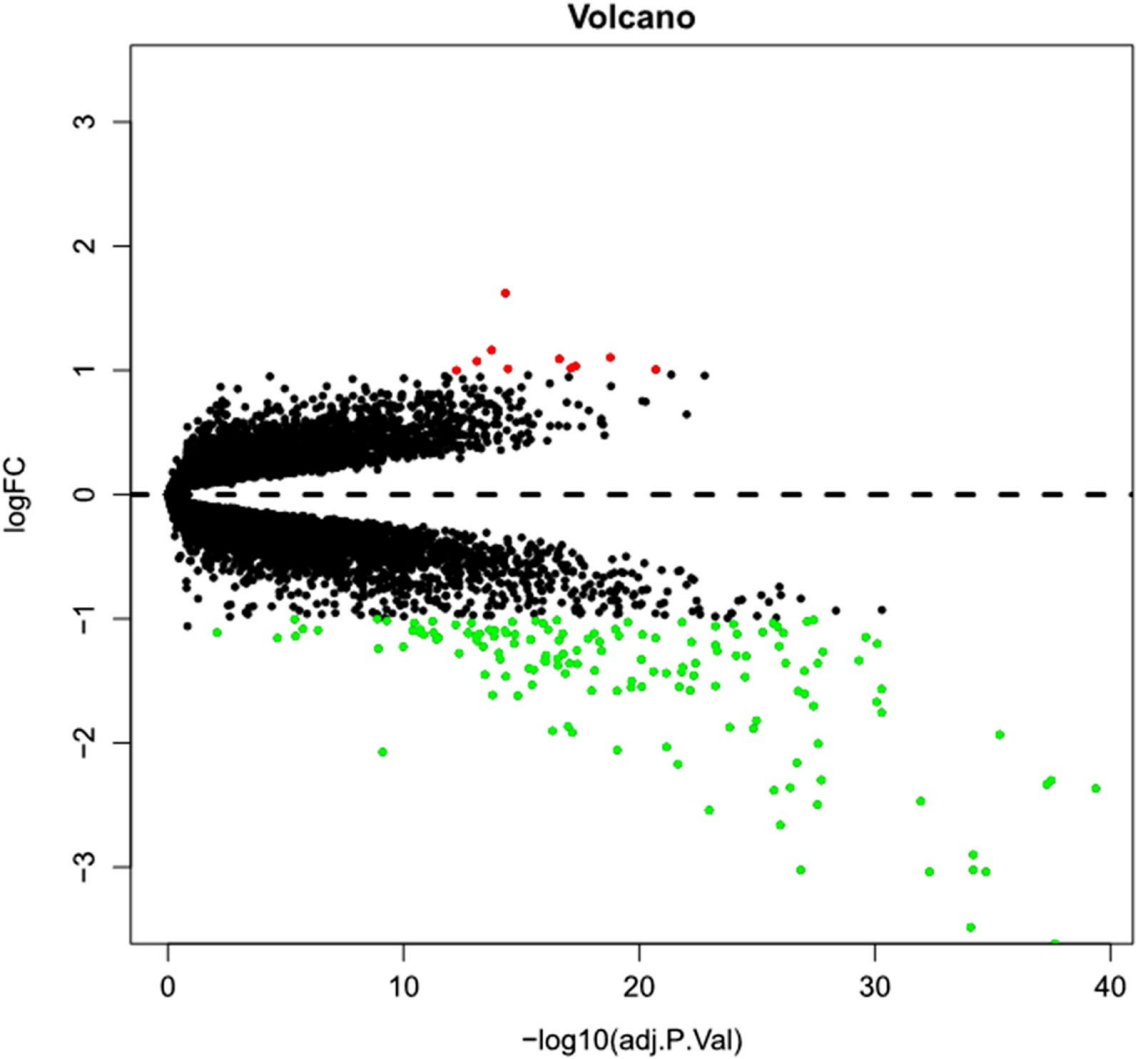
Volcano plot of mRNA differential expression analysis between the normal and TB groups. Red represents significantly upregulated genes, and green represents significantly downregulated genes

TB microarray data (GSE 83456, control: 61, TB: 92) were downloaded from the GEO database. The *limma* package was utilized to perform differential expression analysis on these 153 samples, and 159 DEGs were ascertained (|logFC|> 1 and FDR < 0.05) (Fig. 1). Among these DEGs, 149 were significantly downregulated, and 10 were significantly upregulated.

### GO and KEGG enrichment analyses of TB DEGs

**Fig. 2:**
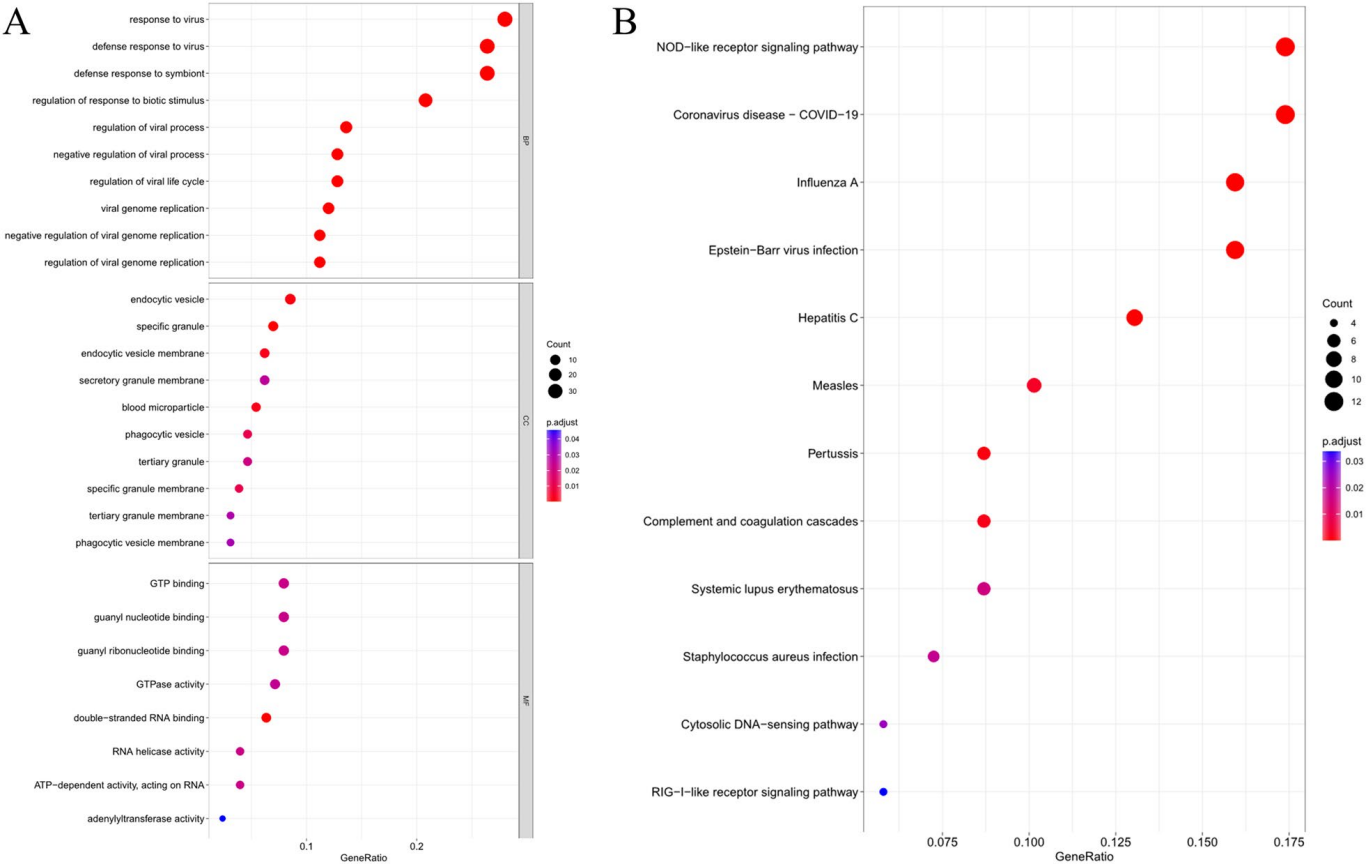
GO and KEGG enrichment analyses of TB DEGs. **A** GO enrichment analysis results; **B** KEGG enrichment analysis results. The size of the bubble represents the number of enriched genes, and the color represents the p-value. The darker the color, the more significant the p-value

Analyses were performed on DEGs with p-values < 0.05, with GO results showing that in the BP module, DEGs were basically enriched in response to virus, regulation of viral process, and defense response to symbiont GO terms. In CC module, they were basically enriched in endocytic vesicle and specific granule GO terms. In MF module, they were basically enriched in GTP binding and guanyl nucleotide binding GO terms, mainly involving virus defense and symbiont defense-related functions (Fig. 2A). KEGG analysis revealed that the majority of these genes exhibited significant enrichment in key pathways such as the NOD-like receptor signaling pathway, innate immune response, and diseases associated with coronaviruses (Fig. 2B).

### Identification of hub genes in TB

**Fig. 3:**
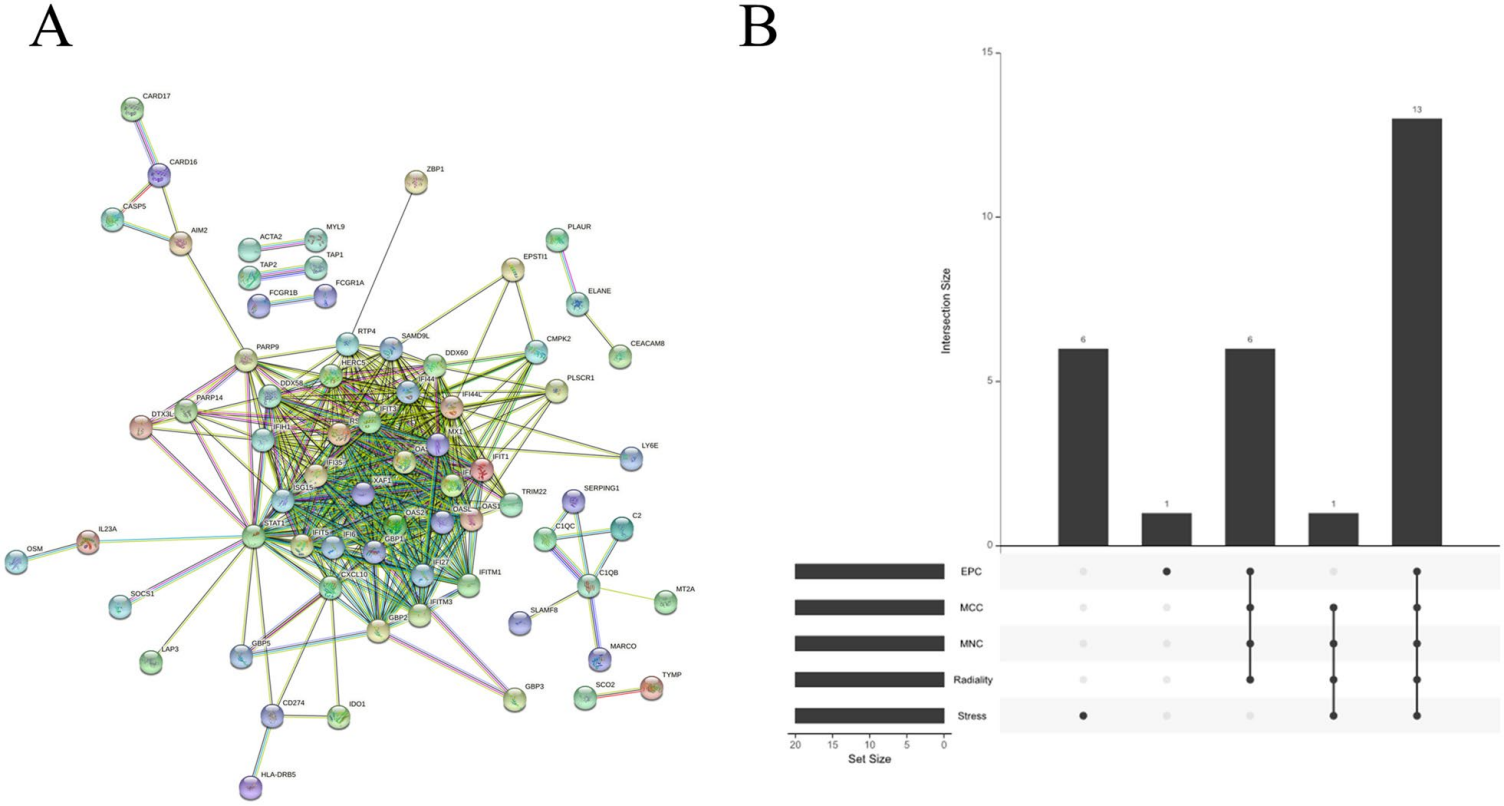
Identification of hub genes in TB. **A** PPI network of the DEGs in the STRING database. Each node represents a protein, and the spiral structure inside the node represents the known three-dimensional structure of the protein. The color of the node represents the score value of the interaction. The lines between nodes represent the interactions between two proteins, and different colors correspond to different interaction types. **B** Upset plot of the hub genes identified using the five algorithms. The x-axis represents the five algorithms, and the y-axis represents the number of genes commonly identified by the five algorithms

STRING database was utilized to build a PPI network of the 159 DEGs, and 139 nodes and 436 edges were obtained with a confidence score > 0.7 (Fig. 3A). Based on the PPI network, the importance coefficients of the DEGs were calculated using five algorithms (EPC, MCC, MNC, Radiality, and Stress) in the Cytoscape software with the cytoHubba plugin, and the top 20 hub genes were selected. Venn diagram was utilized to identify 13 hub genes that were commonly included in all five algorithms (DDX58, IFIT2, IFIH1, RSAD2, IFI44L, OAS2, OAS1, OASL, IFIT1, IFIT3, MX1, STAT1, and ISG15) (Fig. 3B).

### Co-expression network and related functions of the hub genes

**Fig. 4:**
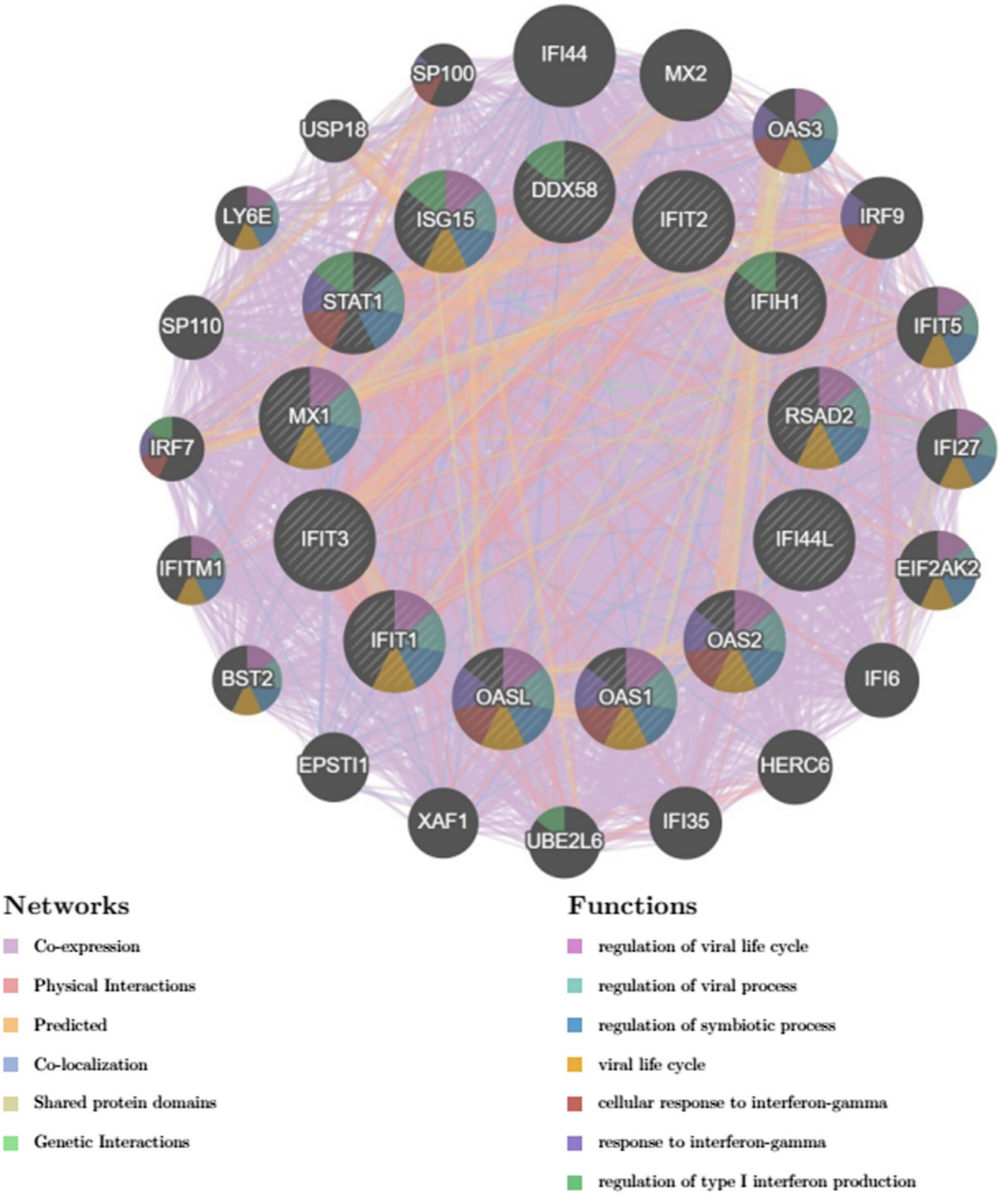
Co-expression network of the 13 hub genes in TB constructed using the GeneMANIA database. The inner layer represents the hub genes, and the outer layer represents the co-expressed genes. The size of the node represents the strength of the correlation, and the color of the line represents the type of interaction

GeneMANIA database was utilized to dissect the co-expression network and related functions of the 13 hub genes (Fig. 4). These genes exhibited a complex co-expression network with a co-expression rate of 81.55%, a physical interaction rate of 12.27%, a co-localization rate of 2.10%, and a predicted rate of 2.24%. They were mainly concentrated in functions related to viral regulation and immune modulation, such as regulation of viral life cycle, regulation of viral process, regulation of symbiotic process, viral life cycle, cellular response to interferon-gamma, response to interferon-gamma, and regulation of type I interferon production.

### Clustering and immune infiltration analysis of hub genes

**Fig. 5:**
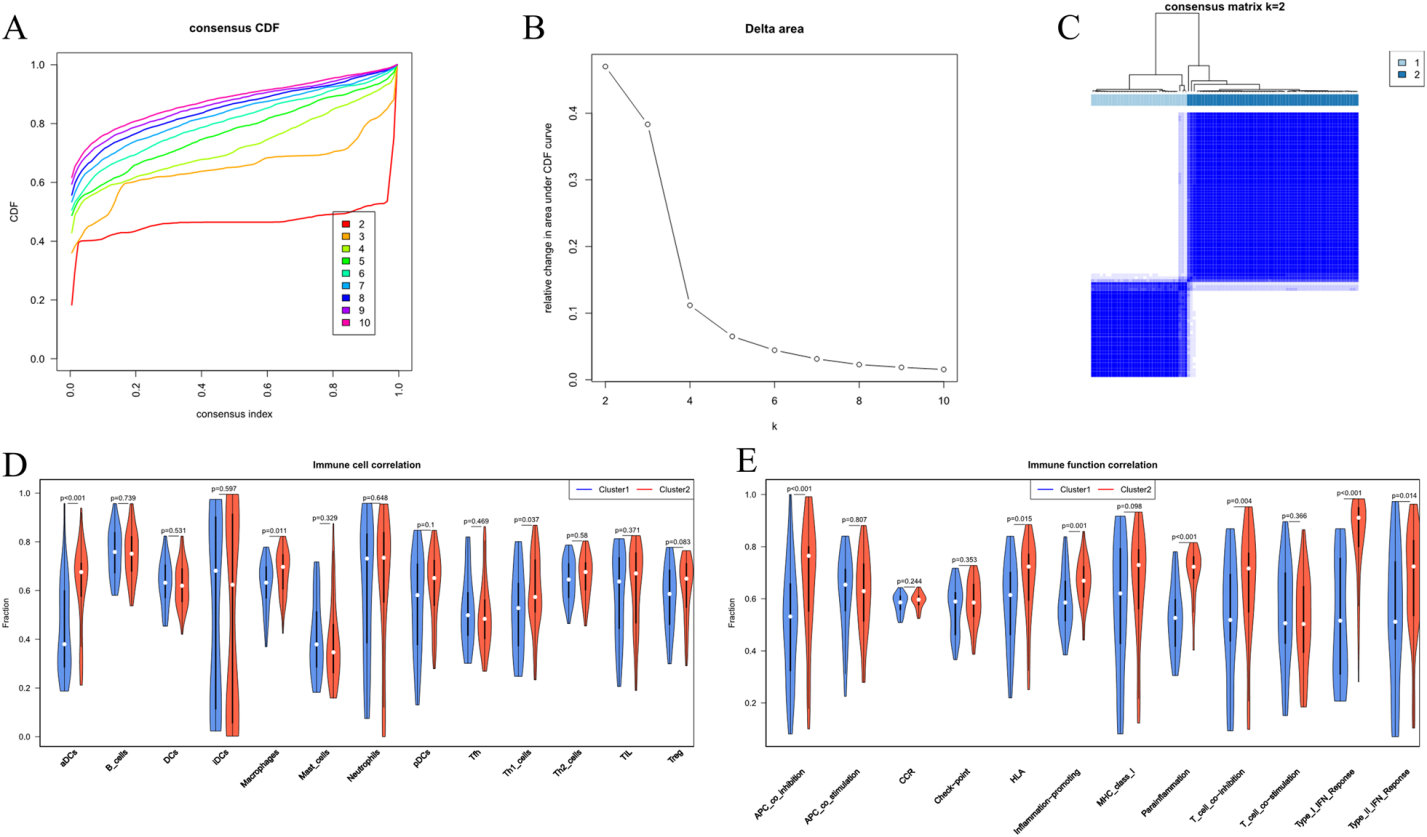
Clustering and immune infiltration analysis of hub genes. **A** Consensus cumulative distribution function (CDF) plot. The different colored curves represent the CDF for different values of k, and the CDF reaches an approximate maximum when k is 2. **B** Relative change in the area under the CDF curve. Each point represents the total area under the CDF curve for a specific value of k, and the appropriate value of k is 2. **C** K-means clustering plot. The rows and columns of the matrix represent the samples, and the consistency matrix is arranged according to the dendrogram above the heatmap, with the bars between the dendrogram and the heatmap indicating the clusters. **D** Analysis of differences in immune cell components in the TB infection subgroups. **E** Analysis of differences in immune function components in the TB infection subgroups

Consensus clustering was manipulated on TB patients based on the expression data of the 13 hub genes. The optimal number of clusters was determined to be 2 grounding on the consensus CDF plot and delta area plot, and a K-means clustering plot was generated (Fig. 5A–C). The immune infiltration levels of each TB patient sample were evaluated using the ssGSEA method. Differences in immune infiltration levels between patients in different subgroups were analyzed, and it was found that the immune cell infiltration levels of aDCs, macrophages, and Th1-cells were higher in subgroup 2 than in subgroup 1 (*P* < 0.05). The immune function scores of APC_coinhibition, HLA, inflammation-promoting, parainflammation, T_cell_co-inhibition, Type_I_IFN_Reponse, and Type_II_IFN_Reponse were tellingly higher in subgroup 2 (*P* < 0.05) (Fig. 5D, E). These results indicated that TB patients in subgroup 2 have a better response to immune therapy.

### Screening of diagnostic biomarkers and model validation

LASSO analysis was performed on the 13 hub genes to screen for 8 key genes, which were IFIT1, IFIT2, IFIT3, IFIH1, RSAD2, OAS1, OAS2, and STAT1. A diagnostic model was constructed from these genes, with the index calculated as follows: index = 0.567 × IFIT3 + 0.0182 × RSAD2-0.1405 × OAS2-1.1373 × IFIT1 + 2.4298 × STAT1 + 0.9271 × OAS1 + 0.0542 × IFIT2 + 18721 × IFIH1.

**Fig. 6:**
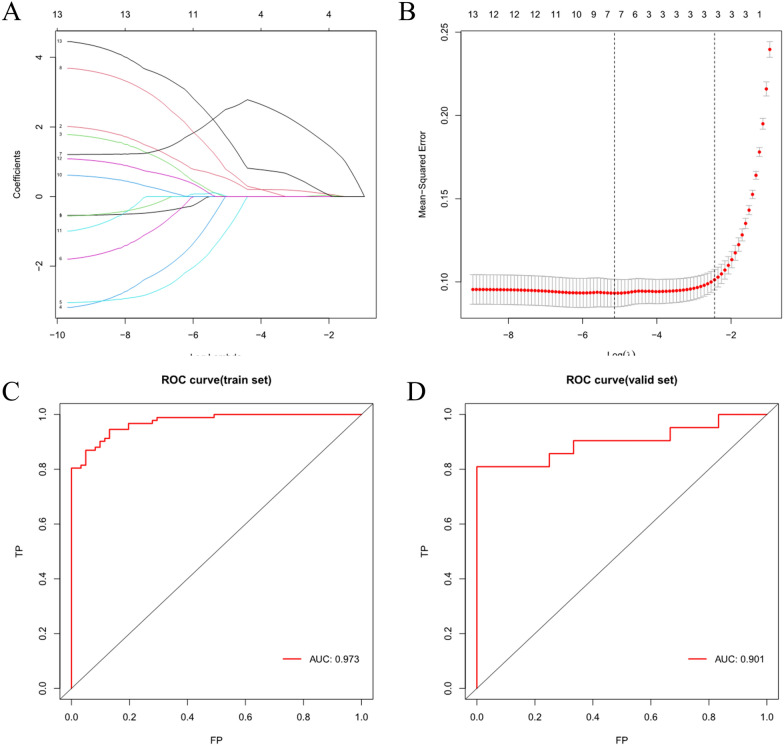
Screening of diagnostic biomarkers and model validation. **A** Coefficient distribution plot generated for the logarithmic sequence (λ) in the LASSO model. **B** LASSO coefficient spectrum of the LASSO Cox analysis. **C** ROC curve analysis of the training set GSE83456. **D** ROC curve analysis of the validation set GSE19444

To validate the accuracy of the model, we depicted an ROC curve using data from training set GSE83456 and calculated the AUC value of the diagnostic model, which was found to be 0.973 (Fig. 6C). The diagnostic ability of the key genes was further verified in the validation set GSE19444, and the diagnostic efficiency of the test set was ascertained to be 0.901 (Fig. 6A–D). The findings strongly indicated that the diagnostic model, constructed using immune-related key genes for TB, exhibited a robust diagnostic performance.

## Discussion

The diagnosis of TB continues to pose significant challenges. Currently, sputum culture serves as the gold standard for TB diagnosis, yet it has long cultivation times, high false-positive rates, and is prone to transmission during the detection process, leading to many problems in clinical use (Chen et al. 2020; Park et al. 2021). The conventional diagnostic approaches are characterized by their time-consuming nature and inefficiency, resulting in potential delays in diagnosing TB patients. This delay, in turn, contributes to the widespread dissemination of the disease and presents a concealed threat to public health. Biomarker sequencing for TB is accurate, time-saving, and cost-effective, and there have been many studies on TB biomarkers. Mpande et al. (2021) inferred the risk of TB by detecting the activity of antigenic T cells in the blood, which can be used to classify different stages of TB infection and develop targeted treatment plans. Wu et al. (2021) employed public datasets, along with bioinformatics analysis and clinical validation, to establish the utility of IRF1 as a novel biomarker for TB diagnosis. To improve the diagnostic efficiency of TB patients, this study screened TB hub genes based on public databases and constructed an 8-gene diagnostic model for TB using LASSO analysis, which had important implications for the diagnosis and management of TB.

By analyzing DEGs between TB patients and healthy individuals and analyzing main functions and related pathways of these genes, we ascertained that these genes were tellingly enriched in viral defense, symbiotic defense, and innate immune response-related functions and pathways. After being infected with Mtb, the body will coordinate multiple signal cascade responses through various pattern recognition receptors to activate multiple innate immune defense functions. The first step in activating innate immune responses during infection is pathogen recognition, and different innate immune cells will use different receptors or receptor combinations to recognize and engulf Mtb (Zhou et al. 2021). The KEGG analysis results from Wen et al. (2022) study reported that a substantial proportion of the DEGs in TB were notably enriched in innate immune pathways, which echoes with the results of this study. Therefore, there is a close relationship between TB and innate immunity.

In order to screen the key genes of TB, this study leveraged STRING database to establish a PPI network of DEGs between TB patients and healthy individuals and identified 13 hub genes using various algorithms in Cytoscape. Subsequently, LASSO analysis was used to screen 8 genes (IFIT1, IFIT2, IFIT3, IFIH1, RSAD2, OAS1, OAS2, and STAT1) to construct a diagnostic model for TB. The IFIT gene family, also known as interferon-induced genes, consists of four members, IFIT1, IFIT2, IFIT3, and IFITM. IFIT family genes are typically expressed at low levels without stimulation, and IFIT is typically transcriptionally induced in response to viral and bacterial infections, participating in the regulation of innate immune responses, and limiting various viruses, stimulating apoptosis of infected cells, and regulating immune responses (Fensterl and Sen 2015). Studies have shown that IFIT1, IFIT2, and IFIT3 are upregulated during latent TB infection, and overexpression of IFIT genes in macrophages leads to a striking increase in key pro-inflammatory cytokines, which can ultimately kill Mtb (Madhvi et al. 2022). OAS, or 2’-5’-oligoadenylate synthetase, is a protein family that encompasses OAS1, OAS2, OAS3, and OASL proteins, which are also a type of interferon-induced gene (Leisching et al. 2018). OAS1 and OAS2 can limit intracellular pathogenic Mtb replication and foster pro-inflammatory cytokine secretion (Leisching et al. 2019). In addition, the pathogenicity and virulence of Mtb strongly induce OASL expression, which can reduce pro-inflammatory cytokine secretion and inhibit the growth and survival of Mtb (Leisching et al. 2020). Cell apoptosis is one of the most important ways for macrophages to clear intracellular Mtb. Yao et al. (2017) showed that elevated levels of phosphorylated STAT1 can impulse the expression of numerous pro-apoptotic genes, thus producing an anti-TB effect. Nevertheless, unphosphorylated STAT1 inhibits macrophage apoptosis, promoting Mtb immune evasion and helping Mtb to persist in infection. Yi et al. (2020) showed that STAT1 is essential for promoting macrophage polarization into M1 polarized macrophages, which can effectively defend against TB infection. In summary, this study built a reliable diagnostic model for TB based on hub genes and identified potential biomarkers for TB.

In conclusion, we used bioinformatics methods to identify 13 hub genes of TB and analyzed their functions and related pathways. Since TB is closely related to immunity, we subsequently screened 8 immune-related genes to construct a TB diagnostic model with good diagnostic performance. This study identified potential therapeutic targets for TB and constructed a reliable diagnostic model for TB. However, although this study was a strict bioinformatics analysis, it still had certain limitations. The diagnostic model for TB was constructed based on data from public databases, and although its predictive performance was good when combined with other datasets, it was not validated in animal experiments or in clinical settings. Future investigations should focus on the validation of the diagnostic performance of the model through multi-center studies.

## Data Availability

Not applicable.
